# Aliovalent Calcium Doping of Yttrium Oxyhydride Thin Films and Implications for Photochromism

**DOI:** 10.1021/acs.jpcc.2c04456

**Published:** 2022-08-19

**Authors:** Diana Chaykina, Ismene Usman, Giorgio Colombi, Herman Schreuders, Beata Tyburska-Pueschel, Ziying Wu, Stephan W. H. Eijt, Lars J. Bannenberg, Gilles A. de Wijs, Bernard Dam

**Affiliations:** † Materials for Energy Conversion and Storage, Department of Chemical Engineering, Delft University of Technology, Van der Maasweg 9, NL-2629HZ Delft, The Netherlands; ‡ Dutch Institute for Fundamental Energy Research, De Zaale 20, NL-5612 AJ Eindhoven, The Netherlands; § Fundamental Aspects of Materials and Energy, Department of Radiation Science and Technology, Faculty of Applied Sciences, Delft University of Technology, Mekelweg 15, NL-2629 JB Delft, The Netherlands; ∥ Storage of Electrochemical Energy, Department of Radiation Science and Technology, Faculty of Applied Sciences, Delft University of Technology, Mekelweg 15, NL-2629 JB Delft, The Netherlands; ⊥ Radboud University, Institute for Molecules and Materials, Heyendaalseweg 135, NL-6525 AJ Nijmegen, The Netherlands

## Abstract

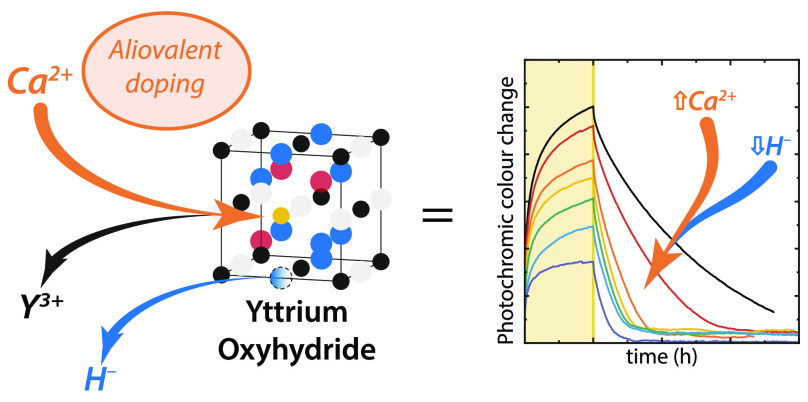

To develop an understanding of the photochromic effect in rare-earth metal oxyhydride thin films (REH_3–2*x*_O_*x*_, here RE = Y), we explore the aliovalent doping of the RE cation. We prepared Ca-doped yttrium oxyhydride thin films ((Ca_*z*_Y_1–*z*_)H_*x*_O_*y*_) by reactive magnetron cosputtering with Ca doping concentrations between 0 and 36 at. %. All of the films are semiconductors with a constant optical band gap for Ca content below 15%, while the band gap expands for compositions above 15%. Ca doping affects the photochromic properties, resulting in (1) a lower photochromic contrast, likely due to a lower H^–^ concentration, and (2) a faster bleaching speed, caused by a higher pre-exponential factor. Overall, these results point to the importance of the H^–^ concentration for the formation of a “darkened” phase and the local rearrangement of these H^–^ for the kinetics of the process.

## I Introduction

Rare-earth metal oxyhydrides (REH_3–2*x*_O_*x*_) are multianion compounds which have gained attention in recent years because they exhibit a photochromic effect.^1^ Thin films of REH_3–2*x*_O_*x*_ (RE = Sc, Y, Nd, Gd, Dy, and Er)^2−6^ are transparent semiconductors which “darken”, or become opaque, upon exposure to light with photon energy greater than their band gap (*E*_incident_ > *E*_g_). When this light is removed, the materials return to their transparent state. Although this so-called photochromic effect is promising for applications such as smart windows, the precise mechanism involved in this effect is yet unknown.

Many properties have been evaluated for their influence on photochromism such as the anion and cation compositions^2,4^ and the defects or inhomogeneities present in the film.^5,7^ One explanation for photochromism has emerged involving a structural rearrangement,^8,9^ perhaps by local diffusion,^10^ to segregate a metallic phase^1,11,12^ of high H^–^ content.^7,11,12^ On the other hand, some suggest the formation of in-gap states by H_2_^13^ or OH^–^^14^ generation as well as H^–^ exchange between phases.^15^

What all these ideas have in common is the displacement of H^–^ by some mechanism for the creation of a metastable “darkened” phase. The mobility of this ion may be enhanced by the creation of anion vacancies throughout the structure, which can be achieved by aliovalent doping. This method involves substituting a cation in the material by one of a lower oxidation state and creating anion vacancies to maintain charge neutrality. Using this method for (perovskite) oxyhydrides^16^ and (rare-earth metal) oxychlorides^17,18^ resulted in improved anion mobility. Here, we dope yttrium oxyhydrides with calcium (Ca^2+^ vs Y^3+^) to assess the effect of this on especially the kinetics of the photochromic effect. Until now, it has been shown that a larger O:H ratio results in a faster bleaching speed, but it is not clear if this is due to the increase in O^2–^ content or the associated anion vacancies.^4^

We show that we can successfully dope yttrium oxyhydride thin films with calcium in the range 0–36%. To compensate for this substitution, the concentration of H^–^ ions appears to be reduced, while the concentration of O^2–^ increases slightly. Above a Ca content of ∼15%, the lattice is strained anisotropically, and the optical band gap expands, which may be related processes. All of the films are photochromic and show a reduction of the photochromic contrast with the substitution of Y for Ca. We propose that the Ca substitution reduces the fraction of octahedral H^–^ and that these entities are important for formation of a “darkened” phase. The bleaching speed is faster as Ca is substituted into the structure due to an increased pre-exponential factor which we attribute to the greater fraction of octahedral vacancies.

## II Experimental Methods

**Figure 1:**
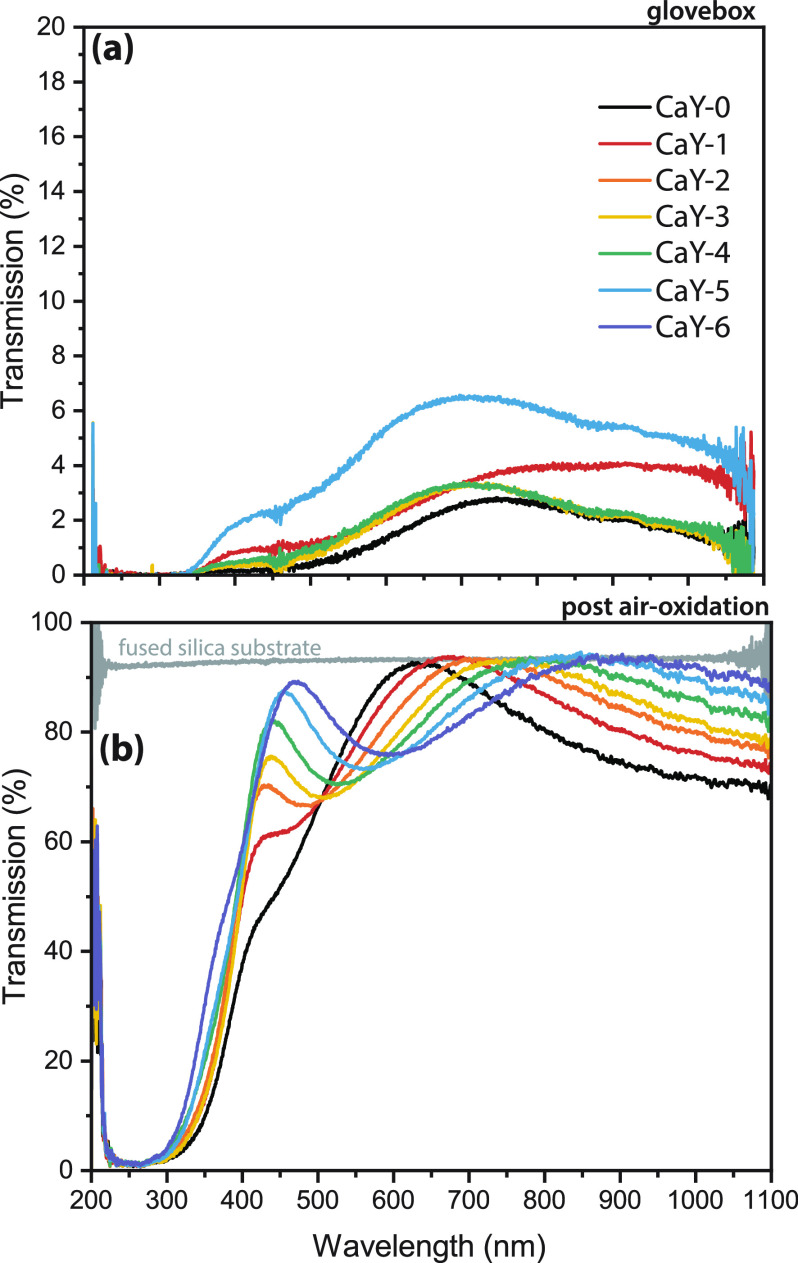
Optical transmission spectra for Ca-doped Y-based thin films (a) in the glovebox before oxidation and (b) post air exposure, showing the dihydride and oxyhydride phases, respectively. Samples CaY-0 to CaY-6 correspond to the Ca contents which vary from 0 to 36% (see Figure 2c).

Ca-doped yttrium oxyhydride thin films ((Ca_*z*_Y_1–*z*_)H_*x*_O_*y*_, ∼ 300 nm) were prepared by reactive magnetron cosputtering of Ca (MaTecK, 99.9%) and Y (Stanford, 99.99%) metal targets onto 10 × 10 mm^2^ quartz plates (MaTecK) at room temperature (∼21 °C) and an Ar/H_2_ (7:1 flow) atmosphere. Following from our previous work on REH_3–2*x*_O_*x*_ thin films (RE = Sc, Y, Nd, Gd, Dy, and Er),^2−5^ the combined Ar/H_2_ deposition pressure (*p*_dep_) affects the as-deposited RE dihydride; we found that if *p*_dep_ is below a critical pressure (p* ∼ 0.4 Pa for Y),^2,4^ the film remains a metallic RE dihydride, but above p*, it forms a semiconducting oxyhydride upon ambient air exposure (Figure 1). Similarly, we find that upon air exposure the as-deposited CaY hydride films become semiconducting, although some of the films already seem to incorporate oxygen when measured in the glovebox before air exposure (glovebox: [H_2_O] and [O_2_] < 0.1 ppm), perhaps due to oxidation from residual gases.^19^ For this work, we used only one *p*_dep_ of 0.5 Pa to survey the effect of Ca doping on the photochromic properties using a range of Ca concentrations (at. %). Co-sputtering was achieved by altering the input DC power to the two targets while keeping a constant total metal flux (Figure S1 and Table SI) of YH_2_ and Ca (Figure S2). Before deposition, the chamber was kept at a base pressure below 10^–6^ Pa.

The composition of the (Ca_*z*_Y_1–*z*_)H_*x*_O_*y*_ films was measured by ion beam analysis using Rutherford backscattering (RBS) and elastic recoil detection (ERD). RBS (ERD) was performed at the DIFFER Ion Beam Facility using a 2.5 MeV ^4^He ion beam at a 0° (75°) incident angle and 170° (23°) scattering angle. The former is used to analyze heavy elements (Ca, Y, O), while the latter is sensitive to light elements (H). For ion beam analysis, the (Ca_*z*_Y_1–*z*_)H_*x*_O_*y*_ thin films were deposited onto glassy carbon substrates (8 × 8 mm^2^) and Si wafers with a native oxide (SiO_2_/Si, 10 × 10 mm^2^). The thickness of these films was ∼150 nm. RBS/ERD data were fitted by using SIMNRA.^20,21^

Dopper broadening positron annihilation spectroscopy (DB-PAS) was used to probe the phase nature of the Ca-doped yttrium oxyhydride thin films. Depth profiles were collected at room temperature by varying the positron (e^+^) implantation energy between 0.1 and 25 keV with the variable energy positron beam (VEP) facility at the Reactor Institute Delft. The energy distribution of the annihilation γ-rays was measured with a high-purity Ge detector (cooled by liquid nitrogen) which has an energy resolution of 1.2 keV. The resulting *S* and *W* parameters were fitted by using the VEPFIT program.

X-ray diffraction (XRD, Bruker D8 Discover) was used to study the effect of Ca doping on the crystal structure of Y-oxyhydride thin films in grazing incident geometry (GI-XRD, incident angle = 2°, primary = 40 mm Goebel mirror with 0.6 mm equatorial slit and 2.5° axial Soller slit, secondary = 0.2° equatorial Soller slit, LynxEye XE detector in 0D mode) and a Cu source. To find the *d* spacing for each peak, they were fit by a double-pseudo-Voigt function considering both *K*_α1_ and *K*_α2_.

First-principles density functional theory (DFT) calculations were conducted with the Vienna *Ab-initio* Simulation Package (VASP)^22,23^ on model structures of (Ca_*z*_Y_1–*z*_)H_3–2*x*–*z*_O_*x*_ (*x* = 0.75, *z* ∼ 3–20%) based on the special quasi-random structures (sQS) of our previous work.^24^ Within the scheme of the projector augmented wave (PAW) method,^25,26^ a plane-wave basis set is used and periodic boundary conditions are applied. Standard frozen core PAW potentials are used, and the H 1s, O 2s2p, Y 4s4p4d5s, and Ca 3s3p4s are treated as valence shells. For each structure, all cell parameters and atomic position are simultaneously optimized employing the PBE generalized gradient approximation for the exchange-correlation functional.^27,28^ After that, the modified Becke–Johnson (mBJ) exchange potentials in combination with L(S)DA-correlation have been used to compute the electronic properties.^29,30^ In all cases, integrations over the Brillouin zone are performed on a 3 × 3 × 3 Γ-centered K-mesh by using a Gaussian smearing of 0.05 eV, and convergence (δ*E* < 0.1 meV) is reached with a kinetic energy cutoff of 850 eV.

Optical transmission spectra were measured by a custom-built setup consisting of an optical fiber spectrometer, a deuterium/quartz tungsten halogen lamp (DH2000-BAL, Ocean Optics B.V.), and a Si array wavelength-dispersive spectrometer (HR4000, Ocean Optics B.V.). Optical band gap energies were determined by using the Tauc method^31^ (Figure S3). Photochromism was measured by illuminating the thin films for 1 h with a narrow wavelength LED (λ = 385 nm, *I* ∼ 75 mW/cm^2^). The average transmission (λ = 450–1000 nm) was plotted with respect to time at room temperature (∼21.5 °C). After illumination, the film was left to “bleach” until its original transparency was recovered. Temperature sweeps were done with the addition of heating at the sample stage (25–55 °C).

## III Results and Discussion

### A Composition and Phase Nature

The compositions of rare-earth metal oxyhydride thin films (made by postoxidation of the as-deposited RE dihydride) have been assessed in our previous work using RBS and ERD, finding that the empirical formula REH_3–2*x*_O_*x*_ (RE = Sc, Y, Gd) describes these materials well.^3^ Starting from the REH_1.9_, upon exposure to air, tetrahedral H^–^ is partly replaced with O^2–^, displacing part of the hydride ions to the octahedral positions.^32^ In the case of aliovalent doping of YH_3–2*x*_O_*x*_ with Ca, we expect that one anionic charge should be removed for every Ca cation substituted. Therefore, we evaluated the compositions of our films in terms of (1) the Ca:Y ratios and (2) the relative change in the anion (O^2–^, H^–^) content.

**Figure 2:**
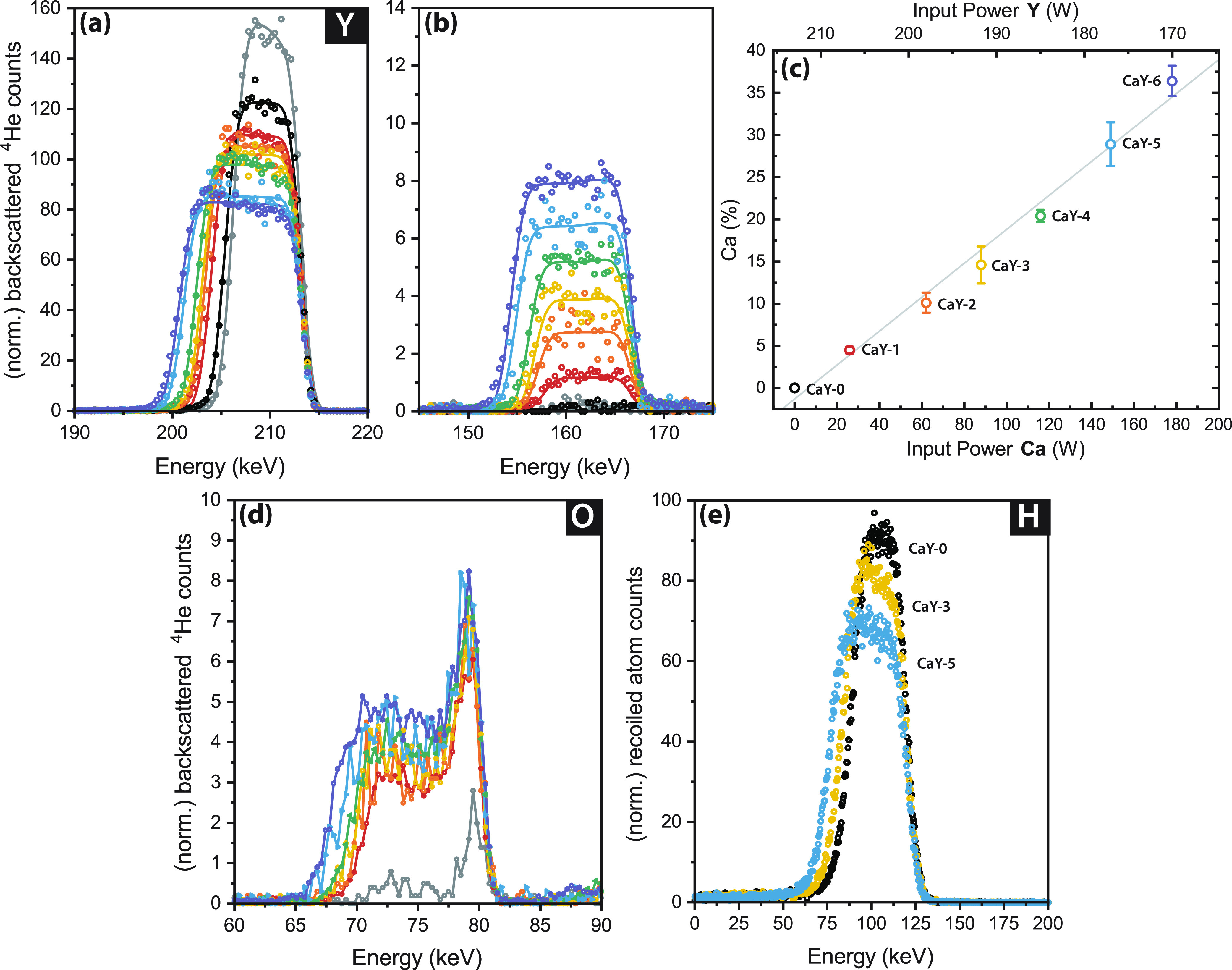
Overview of the compositions of Ca-doped oxyhydride thin films (Ca_*z*_Y_1–*z*_)H_*x*_O_*y*_. For (a) and (b), the lines are from simulations of the composition using SIMNRA. RBS data for (a) yttrium, (b) calcium, and (d) oxygen are shown for YH_1.9+δ_ and a series of oxyhydrides with gradually higher Ca content, where the black points are for CaY-0 (0% Ca) and purple points are for CaY-6 with the most Ca. (c) Ca content calculated from RBS along with the input power to the Ca and Y targets during cosputtering showing the linear relationship. (e) ERD results for hydrogen as more calcium is added to yttrium oxyhydride. All RBS and ERD data are normalized to account for differences in accumulated charge.

Figure 2 shows the results of this composition analysis (full spectra in Figure S4) for YH_1.9+δ_ (reference without Ca or O, gray), YH_3–2*x*_O_*x*_ (reference without Ca, black), and a series of doped Y-oxyhydrides with progressively higher Ca content. The samples are termed CaY-#, with CaY-0 having 0% Ca doping and CaY-6 having the highest Ca content. Comparing first the cations, Figure 2a,b shows the tandem decrease of the Y peak and increase of the Ca peak intensities, suggesting that the cationic ratio was successfully changed by adjusting the DC power to the metal targets during sputtering. Plotting this ratio against the input power during sputtering (Figure 2c) reveals a roughly linear relationship.

For the anions, we qualitatively conclude that the concentration of O does not strongly depend on the Ca/Y ratio (Figure 2d), while the H content decreases more significantly with the addition of Ca (Figure 2e). Apparently, in the given oxidation conditions, this substitution results primarily in the formation of hydride vacancies (instead of oxide vacancies) to maintain charge neutrality.

The phase nature of these films is important to assess because Ca is a highly reactive element with a strong tendency toward oxidation, and one can imagine that phase segregation may occur where Ca creates a secondary phase within the Y-oxyhydride matrix instead of participating in aliovalent doping of the oxyhydride. First, we address the presence of a metallic Ca phase within the oxyhydride. From optical transmission measurements of the Ca_*z*_Y_1–*z*_H_*x*_ films in the glovebox before air exposure (Figure 1a), the addition of Ca did not lower the transmission of the material compared to the undoped YH_∼1.9_. While the substoichiometric YH_∼1.9_ has a transparency window in the visible region,^33^ Ca metal is completely opaque, and the presence of a separate Ca phase would, thus, lower the overall transmission of the material.^34^ After air exposure, the maxima of transmission (caused by thin film interference) touch the transmission of the substrate, meaning that the films have the maximum transparency possible. Had there been a metallic phase, this value would also be lowered. As well, positron annihilation spectroscopy (Figure S5) can be used to rule out the presence of small metallic secondary phases because the positron may annihilate preferentially in metallic centers.^7,35,36^ When a significant amount of Ca metallic domains would have been formed, a larger increase in the positron Doppler broadening *S* parameter is expected than what is observed here.^7,37^ Two samples with either 0 or 20% Ca have nearly the same *S* parameter (Figure S5 and Table SII), suggesting that no metallic phases are present in either case and that their cation vacancy structure is very similar.

Next, it is also possible that CaH_2_ forms a secondary phase within the Y-oxyhydride matrix. However, from the optical transmission measurements shown in Figure 1a, the optical band gap which emerges before air exposure resembles that of the oxyhydride phase (∼2.5 eV) rather than the CaH_2_ phase (∼4.4–5.2 eV).^38,39^ This is likely due to the partial pressure of H_2_ used during sputtering, which may be too low to achieve the CaH_2_ state (Figure S2). The appearance of an optical band gap before air exposure could instead be due to small amounts of O_2_ contamination in the glovebox introduced during sample transfer. As well, the reduction of the H peak from ERD with the addition of Ca suggests that there is no CaH_2_ formation (Figure 2e) because the presence of this phase would not require H to leave the sample to maintain charge neutrality.

**Figure 3:**
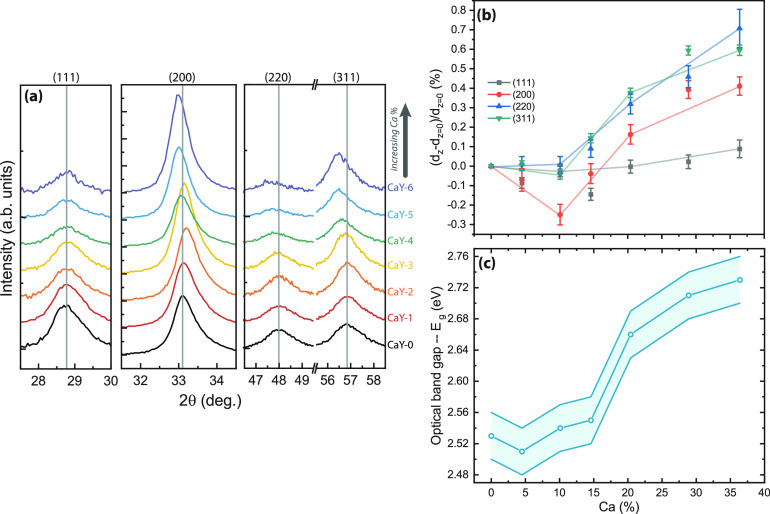
(a) GI-XRD patterns for (Ca_*z*_Y_1–*z*_)H_*x*_O_*y*_ with different Ca content. Vertical gray lines are references for the reflections of the 0% Ca sample. (b) Relative change in *d*-spacing for different reflections as a function of Ca content showing the increase in strain for Ca contents > 15%, and that the strain is not isotropic. Lines are guides for the eye. (c) Change in the optical band gap energy as a function of Ca content, showing an increase above 15% Ca.

Last, we address the possibility of oxidized Ca phases (CaO, Ca(OH)_2_) within the Y-oxyhydride matrix. On the basis of the optical transmission spectra of the films after air exposure (Figure 1b), the transmission and band gap appear to be similar to the oxyhydride phase. Ca oxides and hydroxides have larger band gap energies than the oxyhydride, so they are not visible in the transmission spectra. However, we do not see evidence of any secondary phases in the XRD patterns (Figure 3a). Thus, we conclude that no crystalline oxide or hydroxide phases of calcium form in the film.

Therefore, we propose that the thin films discussed here are single-phase Ca-doped Y-oxyhydrides ((Ca_*z*_Y_1–*z*_)H_*x*_O_*y*_), where Ca substitutes for Y in the lattice (0–36% Ca), and the H^–^ fraction decreases for charge neutrality. There are many examples of single-phase compounds containing both yttrium and calcium such as hydrides,^38,40^ fluorides,^41^ and others.^42,43^ The success of this substitution involving Ca and Y may be attributed to the similar ionic radii of these two elements (Ca^2+^ = 1–1.12 Å, Y^3+^ = 0.9–1.02 Å).^44^ To this long list of single-phase Ca/Y compounds, we suggest to add Ca-doped Y-oxyhydrides for which we find that a stable thin film can be synthesized for a Ca content of at least 0–36%.

### B Structure

We investigate the crystal structures of our films using grazing incident X-ray diffraction (GI-XRD), as shown in Figures 3a and S6. Thin films of YH_3–2*x*_O_*x*_ made by the same methods as used here are face-centered cubic (fcc, *Fm*3̅*m*).^2−4,45^ The GI-XRD patterns in Figure 3a show the expected reflections for a fcc lattice for all samples regardless of Ca doping. However, we could not identify a unique lattice constant for all of these films. We use the vertical gray lines to denote the 2θ reflections of CaY-0 (or YH_3–2*x*_O_*x*_ with 0% Ca) and illustrate how Ca doping influences these peak positions. With increasing Ca content, the (111) reflections remain at the same position as the 0% sample, while the other reflections shift to different 2θ for Ca concentrations >5%.

To better visualize this effect, the relative *d* spacing (with respect to the undoped film) for each reflection is shown in Figure 3b. The *d*_111_ plane is constant for all Ca doping concentrations, while the other planes expand upon increasing Ca doping. Importantly, they do not expand to the same extent, with the *d*_200_ plane expanding by 0.4% while the *d*_220_ and *d*_311_ planes by ∼0.6–0.7% when comparing 0% and 36% Ca. This suggests that the lattice is strained in specific directions by the addition of Ca. This strain deforms the lattice by <1%, such that it is no longer cubic, but rather an orthorhombic lattice where *a* ≠ *b* ≠ *c*. This lattice strain may be caused by the slight difference in the ionic radii of Ca^2+^ and Y^3+^ or the accumulation of vacancy defects.

### C Optical Properties

The optical band gap energies for the films studied here are presented in Figure 3c with respect to their Ca doping concentration. The band gap is quite constant at a value of ∼2.52 eV until a Ca doping level of ∼15%, at which point the band gap expands. This expansion could be explained either by the composition of the thin film, the lattice strain, or a combination of both.

In previous studies, changes in the band gap were attributed to the composition. For example, a higher O:H ratio generally results in a wider band gap.^3,4^ Here, we do not see a sudden change in the O^2–^ content for CaY-4 from RBS (Figure 2d), but it may be that the content of H^–^ decreases enough at this composition to widen the optical band gap. From DFT simulations of 0 and 23% Ca compositions (Figure S7), there is an indication that the H valence band maximum recedes slightly, widening the band gap by about 6%, which is of similar magnitude as observed experimentally. It should be noted that the cationic substitution itself does not seem to affect the band gap because the Ca states are outside the gap. Only changes in the H^–^ composition appear important here.

In addition, lattice strain, as observed in Figure 3b, may cause the band gap to expand. Both the lattice and band gap expansions become significant around the same Ca content of ∼15%. The concept of strain engineering the band gap has been used in many semiconductors^46^ and may play a role here.

### D Photochromism

**Figure 4:**
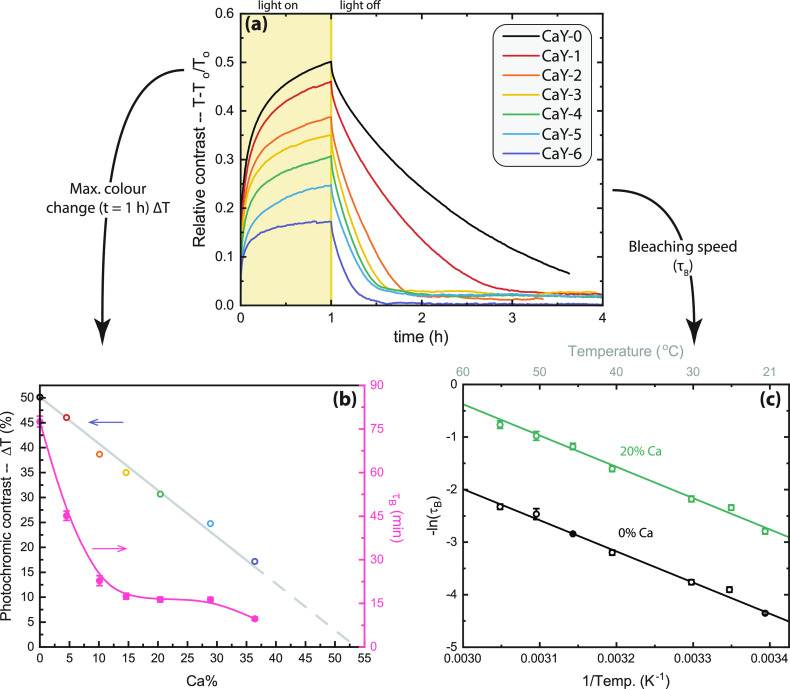
(a) Relative contrast for (Ca_*z*_Y_1–*z*_)H_*x*_O_*y*_ thin films with varying Ca content. When the sample is transparent, the relative contrast is 0. During 1 h of illumination, the contrast increases and later decreases back to 0 when the illumination is stopped. (b) (left *y*-axis) The maximum relative contrast as a function of Ca content. Extrapolation of the linear relationship leads to a prediction of 0% contrast when the Ca doping concentration is ∼54%. (right *y*-axis) The bleaching speed (τ_B_) becomes faster with the addition of Ca, although following a nonlinear relationship. (c) Arrhenius plot of the bleaching speed for (Ca_*z*_Y_1–*z*_)H_*x*_O_*y*_ thin films containing 0 and 20% Ca, showing that differences in bleaching speed are not due to an altered activation energy of the process but are dependent on the pre-exponential factor.

The photochromic properties of the films are measured by illuminating them with a 385 nm LED for 1 h and measuring the average transmission (λ = 450–1000 nm) as a function of time. The two main figures of merit for photochromic materials are the contrast (maximum amount of change in transparency) and the bleaching speed (time required to return to the original transparent state). The data presented in Figure 4a show the relative contrast (Δ*T* (%) = (*T* – *T*_0_)/*T*_0_) instead of the average optical transmission to normalize for slight differences in absolute transmission and show the change in photochromic contrast more clearly. Before illumination, samples are transparent (Δ*T* = 0). This increases as the samples “darken” under illumination (yellow shaded area) and decreases back to the transparent state after illumination.

**Figure 5:**
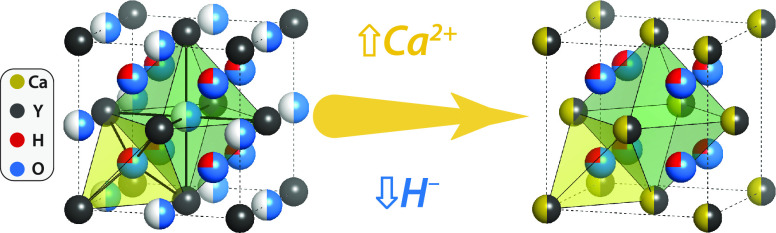
Schematic image of the anion disordered (Ca_*z*_Y_1–*z*_)H_*x*_O_*y*_ unit cell where Ca is a yellow circle, Y is black, H is blue, O is red, and unoccupied sites are white. The disordered nature of the anions is indicated by partial occupation of the interstitial sites, explained in ref (3). When Ca is added, it replaces Y in the lattice, but because the precise position is unknown, this is also represented as partial occupation on all fcc lattice positions. We propose that addition of Ca is compensated by the removal of H^–^ ions from the octahedral sites, such that at a composition of ∼54% Ca (or 50% in this idealized model) all octahedral sites are vacant, and the photochromic contrast is 0% (see text).

The photochromic contrast linearly decreases with the substitution of Y for Ca (Figure 4b), suggesting a direct relationship between the two quantities. Interestingly, the extrapolation of this linear relationship leads to a prediction that no photochromic contrast should be measured for a doping level of ∼54% Ca. We suspect that this linear relationship is actually an indication of the importance of hydride ions in the sample, specifically octahedral hydride ions (Figure 5). While it is true that Y also decreases with the addition of Ca, that alone cannot justify the disappearance of the contrast as there would still be a significant fraction of Y in the film. On the other hand, there can be a large difference in the properties of octahedral versus tetrahedral H^–^, making the decrease in the population of certain H^–^ potentially significant for the observed properties of the material. Not only are the octahedral H^–^ likely the first to leave the structure upon oxidation,^3,32^ but they are often cited as more mobile than tetrahedral H^–^ either due to the lower formation energy for an octahedral H vacancy^14^ or their weaker electrostatic interactions with O^2–^ (more distance).^47^ Having these mobile H^–^ could be an essential ingredient to the formation and dissolution of a “darkened” phase.

The bleaching speed, on the other hand, does not show a linear relationship to the amount of Ca in the samples, although a monotonous decrease can be recognized (Figure 4b). To understand this trend, we studied the temperature dependence of the bleaching speed for a set of 0% Ca and a 20% Ca samples (21–55 °C), which follows an Arrhenius relation (Figure 4c). The bleaching time constant (τ_B_) is derived from first-order kinetics and is related to the concentration of the “dark” species (*c*(*t*)):
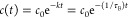
1Combining this with the Lambert–Beer law and the absorption coefficient results in the following:^45^
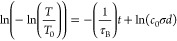
2showing that τ_B_ can be extracted from the slope of the linear fit of a ln(−ln(*T*/*T*_0_)) versus *t* plot (Figures S8 and S9). When the temperature is constant, it is clear from Figure 4a (21.5 °C) that the bleaching speed of the samples becomes faster with increasing Ca content. Considering a range of temperatures, an Arrhenius relationship can be written as
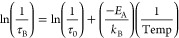
3such that the slope of a ln(1/τ_B_) versus (1/Temp) plot is related to the activation energy (*E*_A_), and the *y* intercept is related to the pre-exponential factor (ln(1/τ_0_) = ln(*k*_0_)). This is displayed in Figure 4c and Table 1; the *E*_A_ values for the two samples are equivalent, while the pre-exponential factors are different. Specifically, the 20% Ca sample, whose τ_B_ at room temperature is ∼79% lower than the 0% Ca sample, shows a pre-exponential factor that is higher by a factor of 5. Therefore, we conclude that the determining factor here for the bleaching speed is the attempt frequency.

**Table 1:** Activation Energies (*E*_A_) and Pre-exponential Factors (τ_0_ = 1/*k*_0_) for Two Samples with Either 0% Ca or 20% Ca Doping in Yttrium Oxyhydride Thin Films^a^

Ca (%)	*E*_A_ (eV)	τ_0_ (s)	*k*_0_ (s^–1^)
0	0.51 ± 0.02	2.3 × 10^–9^	4.4 × 10^8^
20	0.51 ± 0.03	4.2 × 10^–10^	2.4 × 10^9^

a These parameters are derived from the bleaching time constant measured at temperatures between 21 and 55 °C, showing that the effect of Ca doping is to increase the pre-exponential factor.

The rationalization of the attempt frequency in this context is not straightforward because this parameter can be interpreted in many ways. If τ_B_ is related to the conventional diffusion of H^–^ from the “darkened” phase to its original position, the attempt frequency could be explained by the amount of vacant sites, hopping distance, and other factors. Given our previous reasoning on the relation between the Ca doping and the amount of octahedral hydrogen, an explanation involving the increasing amount of octahedral vacancies is the most consistent one and points to a *short-range* diffusion mechanism related to bleaching. On the other hand, for RE oxyhydrides with proven *long-range* H^–^ conductivity (RE = La),^48^ changes in this conductivity were also attributed to the pre-exponential factor rather than the *E*_A_, but citing complex interactions of many H^–^ ions as the source.

## IV Conclusion

In conclusion, we have prepared single-phase aliovalently doped yttrium oxyhydride thin films with Ca (0–36%). These films were made by reactive magnetron cosputtering and air oxidation to achieve the oxyhydride phase. The composition of the cations was verified by RBS, and qualitative analysis of the anions (by RBS and ERD) showed that the O content was largely unaffected by Ca doping, while the H content decreased. As well, the addition of >15% Ca resulted in the appearance of anisotropic lattice strain and a moderate expansion of the optical band gap, two effects which may be related. Importantly, all of these films are photochromic, showing that the photochromic contrast decreases with the addition of Ca, possibly due to the removal of octahedral H^–^ that may be essential for the creation of a “darkened” phase. The bleaching speed became faster due to Ca doping, indicating a potential relation between the bleaching speed, the attempt frequency, and the number of octahedral vacancies. These results point to the importance of local H^–^ diffusion for the understanding of the photochromic mechanism, although a full explanation of this effect should account for other aspects of these materials such as anion disorder and other inhomogeneities.

## Supporting Information Available

Reactive magnetron cosputtering conditions, Tauc plots, Rutherford backscattering spectra, DB-PAS depth profiles, XRD patterns, density of states from DFT simulations, detailed analysis of the photochromic bleaching speed for Ca dependence, and temperature dependence (PDF)
VASP files (ZIP)
