# Correction to “Aliovalent Calcium Doping of Yttrium Oxyhydride Thin Films and Implications for Photochromism”

**DOI:** 10.1021/acs.jpcc.2c06852

**Published:** 2022-10-24

**Authors:** Diana Chaykina, Ismene Usman, Giorgio Colombi, Herman Schreuders, Beata Tyburska-Pueschel, Ziying Wu, Stephan W. H. Eijt, Lars J. Bannenberg, Gilles A. de Wijs, Bernard Dam

The energy axes of the RBS and ERD data (contained in Figures 2a,b,d,e, and S4) were originally underestimated, and the corrected figures appear below and in the Supporting Information. The change is in the conversion from raw data to the energy scale, which was initially converted incorrectly. The rescaled *x*-axis does not change the data conclusions since the assignment of peaks to atoms remains the same and the intensity of the peaks is unaffected. Hence, it has no influence on the calculations and conclusions in the original text.

**Figure 2:**
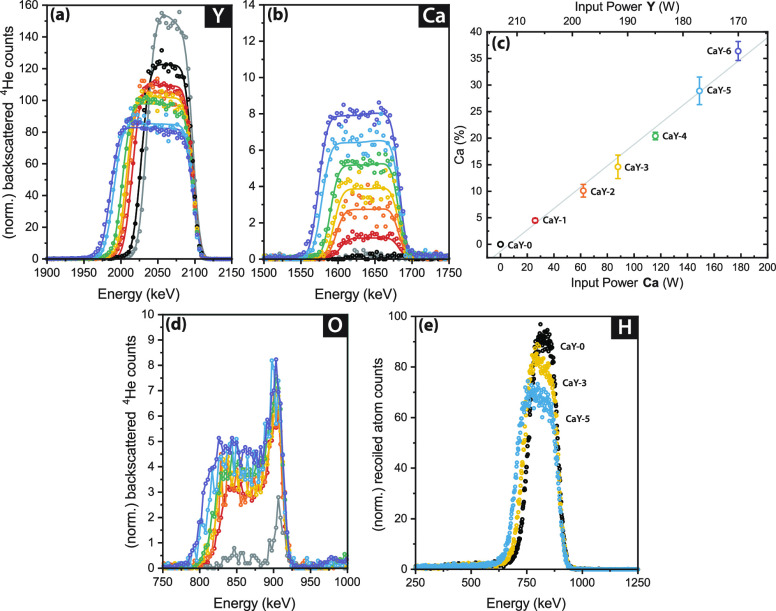
Overview of the compositions of Ca-doped oxyhydride thin films (Ca_*z*_Y_1–*z*_)H_*x*_O_*y*_. For (a) and (b), the lines are from simulations of the composition using SIMNRA. RBS data for (a) yttrium, (b) calcium, and (d) oxygen are shown for YH_1.9+δ_ and a series of oxyhydrides with gradually higher Ca content, where the black points are for CaY-0 (0% Ca) and the purple points are for CaY-6 with the most Ca. (c) The Ca content calculated from RBS along with the input power to the Ca and Y targets during cosputtering showing the linear relationship. (e) ERD results for hydrogen as more calcium is added to yttrium oxyhydride. All RBS and ERD data are normalized to account for differences in accumulated charge.

## Supporting Information Available

Reactive magnetron cosputtering conditions, Tauc plots, corrected Rutherford backscattering spectra, DB-PAS depth profiles, XRD patterns, density of states from DFT simulations, and detailed analysis of the photochromic bleaching speed for Ca dependence and temperature dependence (PDF)

