# Supplementary material for: Correction to “Aliovalent Calcium Doping of Yttrium Oxyhydride Thin Films and Implications for Photochromism”
Source: J Phys Chem C Nanomater Interfaces. 2022 Oct 24;126(43):18586. doi: 10.1021/acs.jpcc.2c06852 (PMC9639169; doi:10.1021/acs.jpcc.2c06852)
Supplement: Supplementary file 1 — jp2c06852_si_001.pdf [file jp2c06852_si_001.pdf]

Supporting information for:

Aliovalent Calcium Doping of Yttrium Oxyhydride Thin Films and Implications for Photochromism

Diana Chaykina*,[1,a] Ismene Usman,[1] Giorgio Colombi,[1] Herman Schreuders,[1] Beata Tyburska-Pueschel,[2] Ziying Wu,[3] Stephan Eijt,[3] Lars J. Bannenberg,[1,4] Gilles A. de Wijs,[5] and Bernard Dam[1]

[1)] *Materials for Energy Conversion and Storage, Department of Chemical Engineering, Delft University of Technology, Van der Maasweg 9, NL-2629HZ Delft, The Netherlands*

[2)] *Dutch Institute for Fundamental Energy Research, De Zaale 20, NL-5612 AJ Eindhoven, The Netherlands*

[3)] *Fundamental Aspects of Materials and Energy, Department of Radiation Science and Technology, Faculty of Applied Sciences, Delft University of Technology, Mekelweg 15, NL-2629 JB Delft, The Netherlands*

[4)] *Storage of Electrochemical Energy, Department of Radiation Science and Technology, Faculty of Applied Sciences, Delft University of Technology, Mekelweg 15, NL-2629 JB Delft, The Netherlands*

[5)] *Radboud University, Institute for Molecules and Materials, Heyendaalseweg 135, NL-6525 AJ Nijmegen, The Netherlands*

[a)] Electronic mail: d.chaykina@tudelft.nl

CONTENTS

I. Co-sputtering of Ca and Y S3

II. Tauc plots S7

III. RBS S8

IV. DB-PAS S9

V. XRD S11

VI. DFT S12

VII. Bleaching curves S13

VIII. PCE for Arrhenius S14

References S15

I. CO-SPUTTERING OF Ca AND Y

To deposit Ca-doped yttrium oxyhydride thin films, Ca and Y metal targets were co-sputtered with a reactive gas mixture (Ar/H_2 = 7:1, p_{dep} = 0.5 Pa). The amount of Ca-doping in the sample was controlled by altering the input power to the two metal targets.

To do this, we first established an atomic flux (ϕ_M) vs. input DC power relationship for the two targets, which is expected to be linear (Fig. S1). We sputtered Y and Ca individually (Ar/H_2 = 7:1, p_{dep} = 0.5 Pa), at different input power, and measured the thickness of the films by profilometry. Using the film thickness deposited for a known period of time, we can determine the sputtering rate for a particular input power (r, cm/s), and then the flux by:

$$\phi_M = \frac{N_A r d}{m} \tag{1}$$

where ϕ_M is the flux of material M, N_A is Avogadro's number, d is the density of material M, and m is the molar mass of the material M.

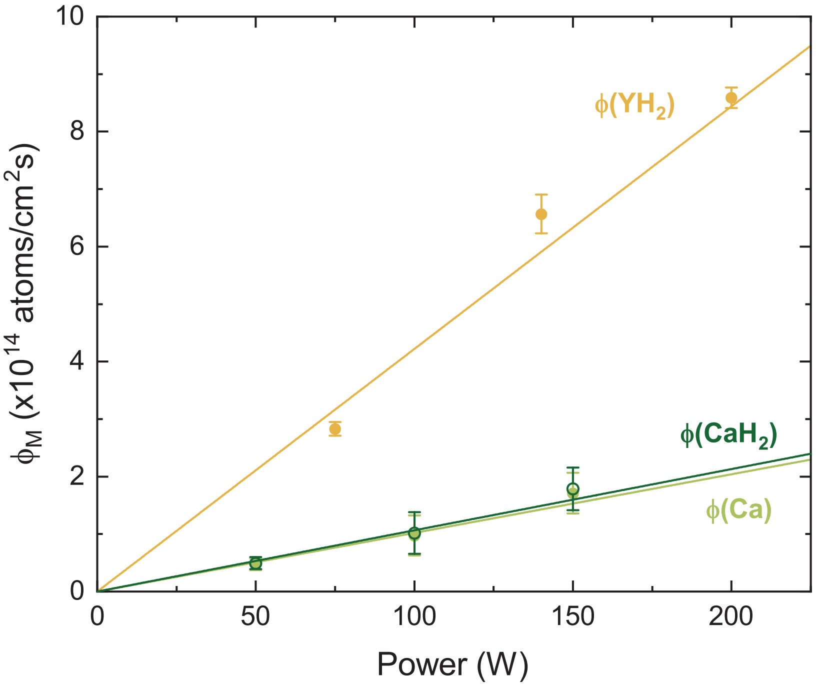

Figure S1: The relationship between the flux (ϕ_M) and input DC power for Y- and Ca-based targets during sputtering with a deposition pressure of 0.5 Pa (7:1 Ar/H_2). For Ca-based films, the data was calculated considering both Ca (light green) and CaH_2 (dark green) as the sputtered species, but this did not influence the slope significantly.

To use Eq. 1, it is necessary to know the identity of the sputtered species since that determines the density and molar mass. For Y-based films, it is known that before air-exposure, the as-deposited film is $YH_{1.9+\delta}$[1–3]. However, the film oxidises to the oxyhydride phase before its thickness is measured. Therefore, the flux obtained in Figure S1 is slightly underestimated since the oxidation of the as-deposited dihydride will lead to a slight expansion[1,2]. However, this is still a valid *estimation* for the necessary sputtering conditions to achieve Ca-doped films.

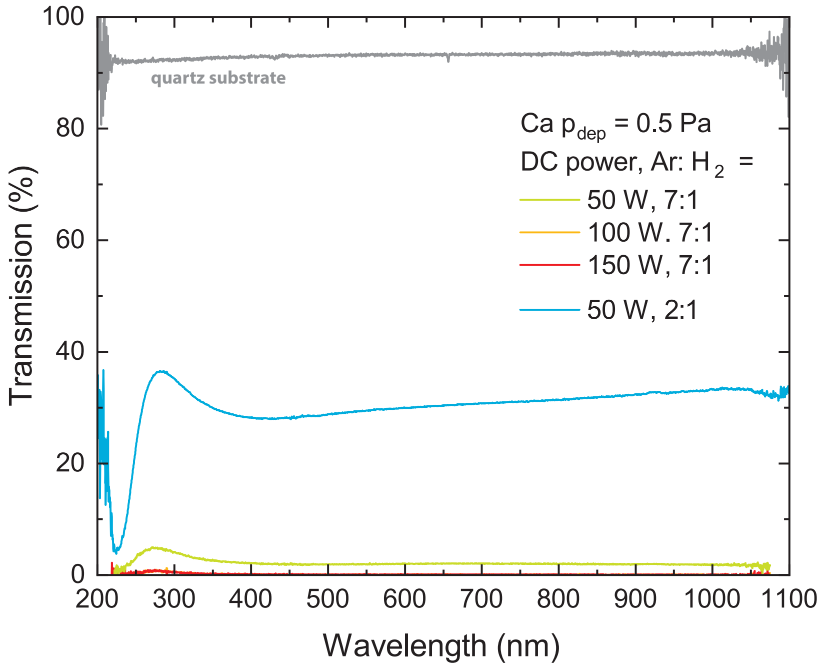

Figure S2: Transmission spectra for four Ca-based films with a reference for the maximum transmission possible given by the quartz substrate. The green (50 W), orange (100 W), and red (150 W) curves are for samples made by sputtering a Ca target at 0.5 Pa (Ar/H_2 = 7:1). The low transmission is indicative of Ca metal. The blue curve (50 W) is for a sample sputtered at a higher H_2 partial pressure (0.5 Pa, Ar/H_2 = 2:1), and may represent CaH_2.

In terms of the Ca-based films, whether Ca or CaH_2 is being deposited does not influence the flux/power estimation significantly (Fig. 1). Despite that, we show evidence in favour of the idea that Ca is deposited, perhaps with a small amount of H incorporation. Ca-based films were sputtered at 50, 100, and 150 W and a pressure of 0.5 Pa (Ar/H_2 = 7:1). The transmission spectra of these films were measured in the glovebox and are shown in Figure S2. All three films show very low transmission, which does not resemble CaH_2.

In general, Ca is expected to be an opaque metal, while CaH_2 is a semiconductor with a band gap around either 2.5 eV for the cubic phase or, more commonly, 4.4-5.2 eV for the orthorhombic phase[4,5]. It may be that the sputtering rate of Ca is too fast, and the partial pressure of H_2 is too low to create the dihydride phase under the conditions we use for sputtering the Ca-doped yttrium oxyhydride thin films.

A previous work on *in situ* deposition of (orthorhombic) CaH_2 by sputtering used a much higher H_2 partial pressure than our methods ($Ar:H_2 = 1:2$), and an RF power source[5]. In Figure S2 we sputtered one film with a much higher H_2 partial pressure and achieved a film with a band gap of $\sim$ 4.0 eV, likely CaH_2. Thus, it may be possible to sputter CaH_2, if a sufficiently high H_2 partial pressure is used. However, we suppose that the Ca deposited under our standard conditions is mostly Ca, although the exact composition does not affect the flux estimation significantly. We note that the Ca films had to be covered by a thin layer of metal with a known thickness to protect the Ca films from oxidation while profilometry measured were performed.

Producing films with different Ca-doping concentrations requires balancing the input power given to the Y and Ca targets, while keeping the total flux at the same value. The total flux (ϕ_{total}) is the summation of the YH_2 and Ca fluxes ($\phi_{YH_2} + \phi_{Ca}$), and we set this value to 9×10^{14} atoms/cm^2-s. This value for the total flux is chosen for practical reasons, i.e., so that the resultant necessary input power to the targets is not too high or too low. The calculated input power to the targets is shown in Table SI.

Table SI: Calcium and yttrium were co-sputtered by reactive magnetron sputtering to produce Ca-doped $YH_{3-2x}O_x$ thin-films. The total flux (ϕ_{total}) was kept constant at a value of 9×10^{14} atoms-cm/s by adjusting the input power to each target (based on the desired Ca:Y ratio).

Sample ID	Power Y (W)	Power Ca (W)
CaY-0	213	0
CaY-1	207	26
CaY-2	198	62
CaY-3	192	88
CaY-4	185	116
CaY-5	177	149
CaY-6	170	178

II. TAUC PLOTS

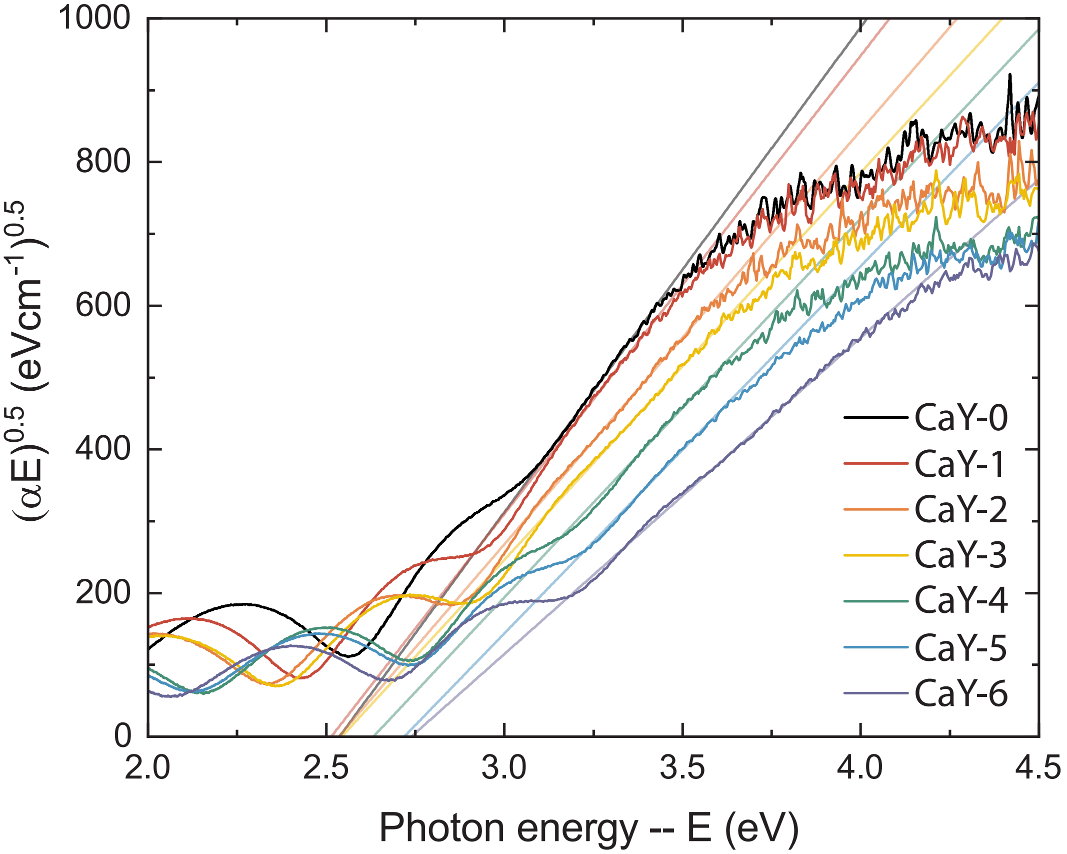

Figure S3: Optical transmission spectra for Ca-doped Y-oxyhydride thin films were converted to Tauc plots and fitted with a linear regression extrapolated to the x-axis. The x-intercept indicates the optical band gap of the material. It is clear that the band gap expands slightly starting with sample CaY-4, which has a Ca-content of ∼15%.

III. RBS

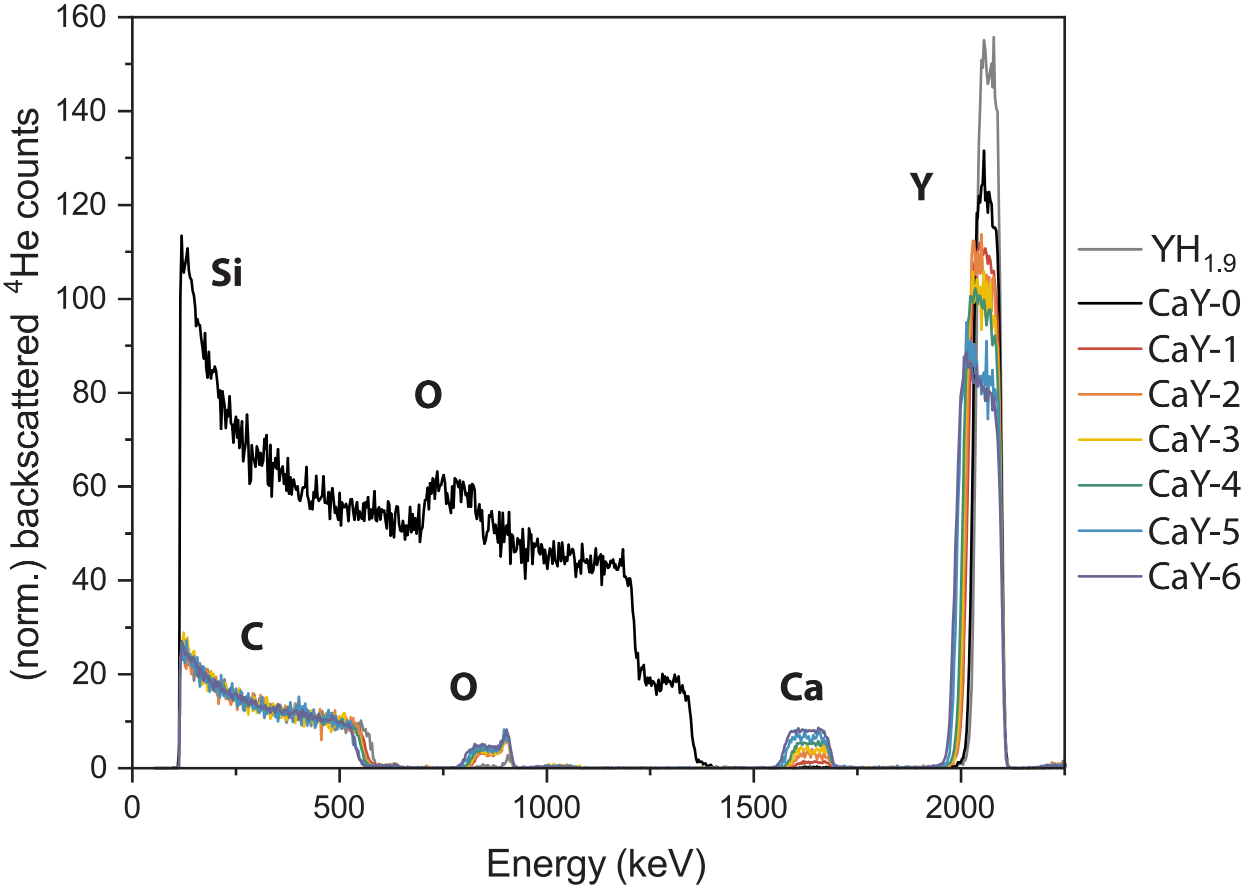

Figure S4: Rutherford backscattering (RBS) spectra for a series of samples with increasing Ca-content. All samples containing Ca (CaY-1 to CaY-6) were measured on glassy carbon substrates, while the undoped Y-oxyhydride (CaY-0) was on a substrate of SiO_2/Si which not only obscured the O peak, but has a different background offset due to the underlying silicon.

IV. DB-PAS

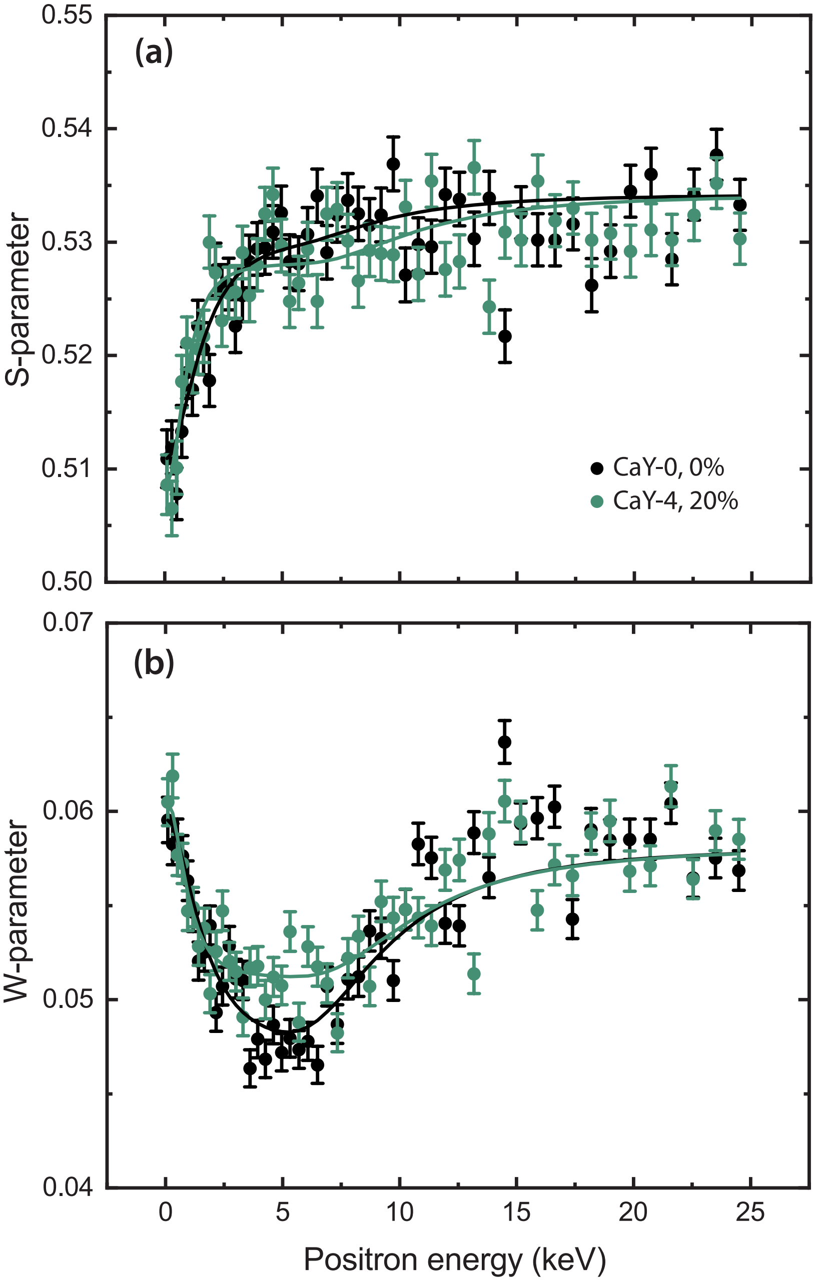

Figure S5: The (a) S-parameter and (b) W-parameter from positron annihilation depth profiles of CaY-0 (black) and CaY-4 (green). The data were fitted by VEPFIT, represented by the solid lines. The S-parameter is generally related to point defect structure and electronic structure, while the W-parameter is influenced by changes in the local chemical environment of the positron annihilation site.

Table SII: The S- and W-parameters derived from DB-PAS depth profiles for 0% and 20% Ca in $(Ca_zY_{1-z})H_xO_y$ thin films. The change in the two parameters is shown as a percentage. From this, we conclude that: (1) the cation vacancy structure in these two materials is similar, (2) there are no metallic centres in the Y-oxyhydride matrix, and (3) the local chemical environments of the positron annihilation sites of the two materials are slightly different. The last point is explained by the wider optical band gap of the 20% Ca film, and the slightly higher O:H ratio as some of the H^- is removed.

Ca (%)	S-parameter	W-parameter
0	0.5302 ± 0.0003	0.0476 ± 0.0002
20	0.5282 ± 0.0003	0.0512 ± 0.0002
	-0.4 %	+7.6%

V. XRD

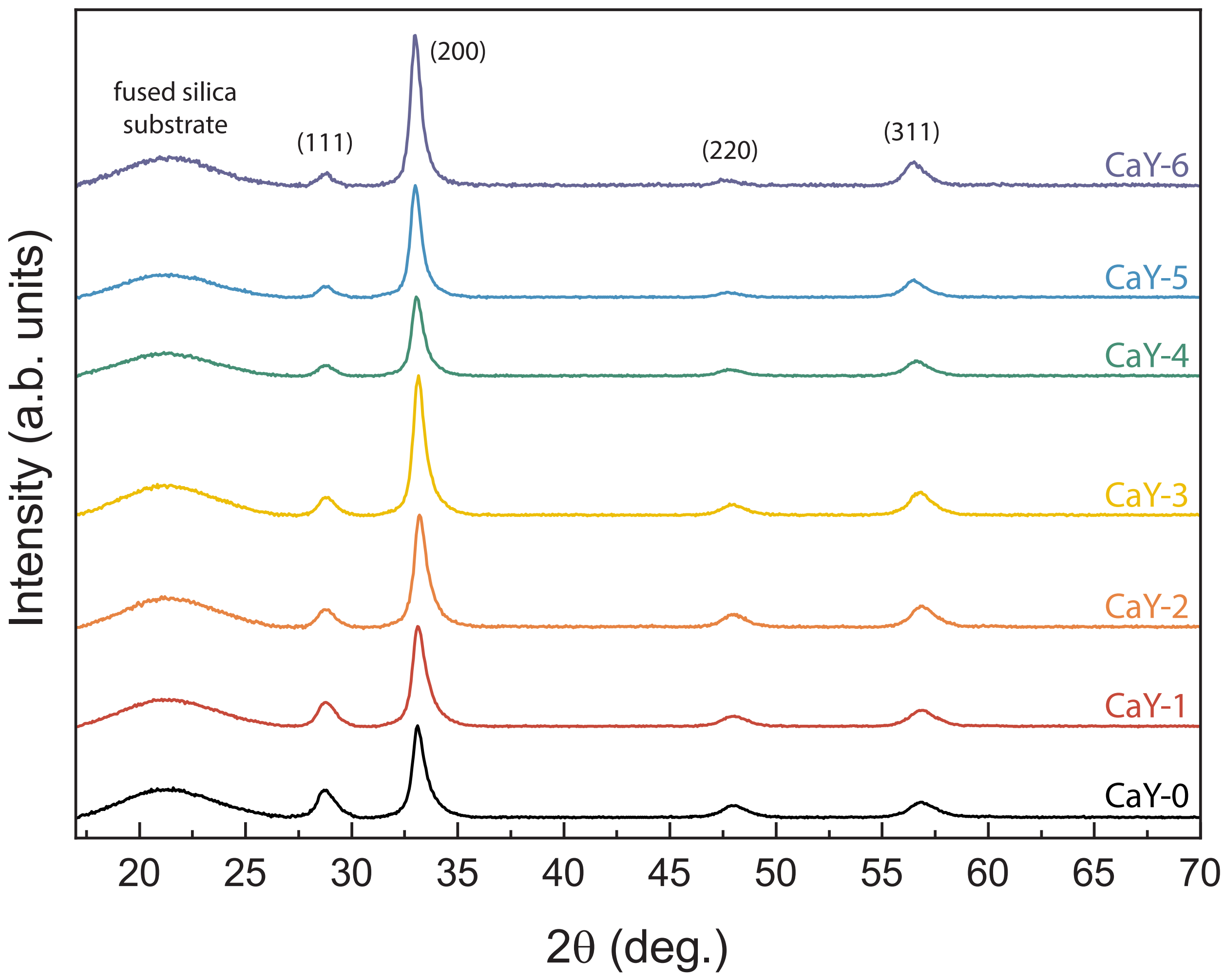

Figure S6: Full grazing incident X-ray diffraction patterns for samples with increasing Ca content from CaY-0 (0%) to CaY-6 (36%).

VI. DFT

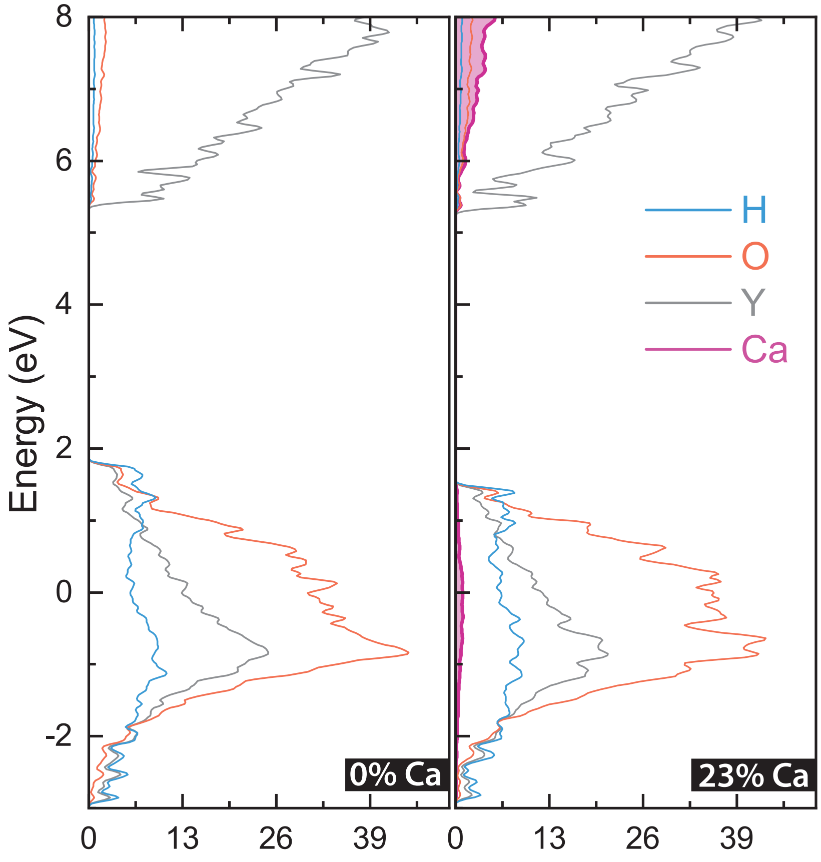

Figure S7: Simulated density of states for two compositions of Ca: (left) 0% and (right) 23%. The contributions of H (blue), O (red), Y (grey), and Ca (pink) to the band structure are shown.

VII. BLEACHING CURVES

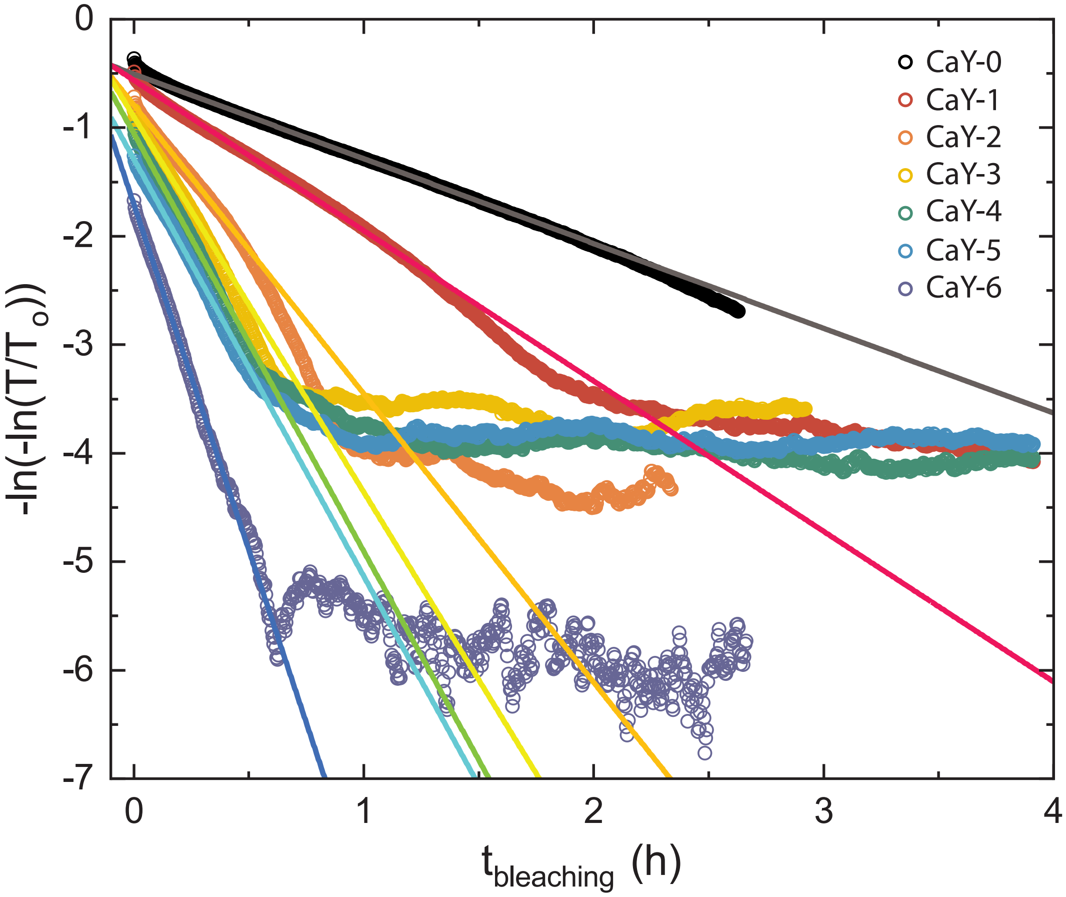

Figure S8: The bleaching speed (τ_B) for samples with increasing Ca-content are derived from the slope of this plot based on first-order kinetics. It is clear that when the Ca-content increases, the bleaching speed becomes faster.

VIII. PCE FOR ARRHENIUS

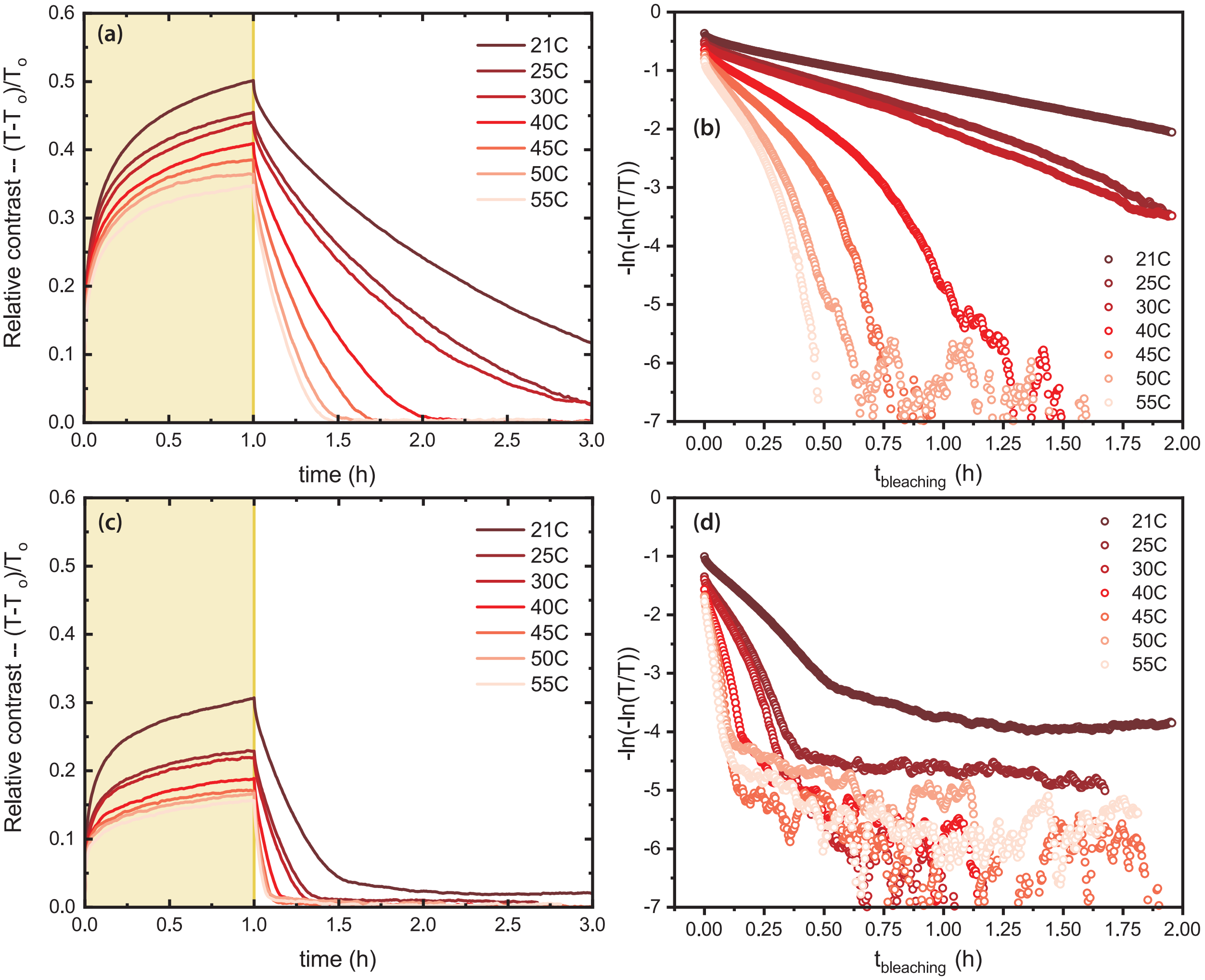

Figure S9: The photochromic effect was measured for a set of (a) 0% Ca and (c) 20% Ca films at different temperatures between 21.5 and 55°C. The bleaching time constants (τ_B) for each temperature were derived from the linear fits for (b) 0% Ca and (d) 20% Ca.

REFERENCES

[1] G. Colombi, T. De Krom, D. Chaykina, S. Cornelius, S. W. H. Eijt, and B. Dam, "Influence of cation (RE = Sc, Y, Gd) and O/H anion ratio on the photochromic properties of REO_xH_{3-2x} thin films," ACS Photonics **8**, 709–715 (2021).

[2] S. Cornelius, G. Colombi, F. Nafezarefi, H. Schreuders, R. Heller, F. Munnik, and B. Dam, "Oxyhydride nature of rare-earth-based photochromic thin films," Journal Physical Chemistry Letters **10**, 1342–1348 (2019).

[3] D. Chaykina, T. de Krom, G. Colombi, H. Schreuders, A. Suter, T. Prokscha, B. Dam, and S. Eijt, "Structural properties and anion dynamics of yttrium dihydride and photochromic oxyhydride thin films examined by in situ μ^+SR," Physical Review B **103**, 224106 (2021).

[4] H. Mizoguchi, S. Park, T. Honda, K. Ikeda, T. Otomo, and H. Hosono, "Cubic fluorite-type CaH_2 with a small bandgap," Journal of the American Chemical Society **139**, 11317–11320 (2017).

[5] M. Gonzalez-Silveira, R. Gremaud, H. Schreuders, M. J. van Setten, E. Batyrev, A. Rougier, L. Dupont, E. G. Bardaji, W. Lohstroh, and B. Dam, "In-situ deposition of alkali and alkaline earth hydride thin films to investigate the formation of reactive hydride composites," Journal of Physical Chemistry C **114**, 13895–13901 (2010).
